# A Modified Multivariable Complexity Measure Algorithm and Its Application for Identifying Mental Arithmetic Task

**DOI:** 10.3390/e23080931

**Published:** 2021-07-22

**Authors:** Dizhen Ma, Shaobo He, Kehui Sun

**Affiliations:** School of Physics and Electronics, Central South University, Changsha 410083, China; madizhen54810@163.com (D.M.); kehui@csu.edu.cn (K.S.)

**Keywords:** complexity, PCA, multivariable, permutation entropy, chaotic series, EEG signal

## Abstract

Properly measuring the complexity of time series is an important issue. The permutation entropy (PE) is a widely used as an effective complexity measurement algorithm, but it is not suitable for the complexity description of multi-dimensional data. In this paper, in order to better measure the complexity of multi-dimensional time series, we proposed a modified multivariable PE (MMPE) algorithm with principal component analysis (PCA) dimensionality reduction, which is a new multi-dimensional time series complexity measurement algorithm. The analysis results of different chaotic systems verify that MMPE is effective. Moreover, we applied it to the comlexity analysis of EEG data. It shows that the person during mental arithmetic task has higher complexity comparing with the state before mental arithmetic task. In addition, we also discussed the necessity of the PCA dimensionality reduction.

## 1. Introduction

The complexity measurement algorithms and their applications are the current research hotspots in the field of nonlinear signal processing. It is widely used to evaluate the irregularities of time series obtained from various systems, such as EEG signals [1,2,3], ECG signals [4,5], walking stride interval signals [6], stock fluctuations [7] and weather prediction [8]. At the meantime, many researchers have conducted in-depth analysis on the complexity of chaotic systems [9,10,11,12]. These researches give us a deeper understanding about the characteristics of chaotic systems.

In order to measure the complexity of time series, many complexity algorithms were proposed, such as the approximate entropy (ApEn) [13], sample entropy (SampEn) [14], fuzzy entropy (FuzzyEn) [15], dispersion entropy (DE) [16] and permutation entropy (PE) [17]. These algorithms have a variety of different advantages. For instance, ApEn does not need to perform binarization or other coarse-grained processing on the time series, and only needs a shorter sequence to estimate a more reliable approximate entropy value. SampEn has a powerful ability to quantify sequence complexity, but the calculation speed is slow. In order to meet the needs under different conditions, researchers have made many improvements on their basis. For example, Coast et al. [18] proposed the multi-scale coarse graining process and designed the multiscale entropy to analyze the time series on multiple time scales. Subsequently, a variety of different multi-scale complexity algorithms were proposed, such as multiscale SampEn (MSE) [19], multiscale fuzzy entropy (MFE) [20], multiscale dispersion entropy (MDE) [21] and multiscale permutation entropy [22]. Obviously, designing of entropy algorithms and complexity measure methods for nonlinear time series developed greatly. Meanwhile, among the algorithms mentioned above, PE algorithm has the characteristics of good anti-noise ability and fast calculation speed. Thus, it is an effective algorithm and was commonly used to analyze the complexity of one-dimensional time series.

Moreover, there exist many high-dimensional systems and multivariate data in the applications. For example, the EEG signals have 21 channels, generally. Thus, it is necessary to design multivariate complexity measures. Until now, there are several different kinds of multivariate complexity measures are proposed, such as multivariate SampEn [23] and multivariate FuzzyEn [24]. Moreover, Ahmed M.U. et al. [25] proposed multivariate multiscale entropy for multivariate data. It adapts to biological and physical systems with the characteristics of multivariate, correlation and noise in the real world, and reveals the long-range correlation between channels. Later, Francesco C.M. et al. [22] proposed multivariate multiscale PE to analyze EEG signals of Alzheimer’s patients. However, in the absence of noise, this algorithm has a lower complexity value in low-frequency signals. In order to preserve the information of the original multi-dimensional data to a large extent, to save the storage space and to speed up the calculation, some researchers used the principal component analysis (PCA) method for data dimensionality reduction [26,27,28]. Marisa M. et al. [26] used the PE algorithm to classify the data set processed by PCA and showed obvious advantages. However, its results did not show the superiority of taking multiple principal components over a single principal component. However, PCA provides a way to shrink the dimension of the data and keep as much as information of the multivariate data.

At present, it is very interesting to employ the linear or non-linear methods to classify EEG signals in different states. Some researchers used neural network methods to classify EEG in different states [29,30,31,32,33], such as emotion recognition [30], fatigue detection [31], epilepsy prediction [32,33], and some other diseases. Furthermore, other researchers achieved the purpose of state classification by calculating the complexity of EEG signals [12,34]. In 2015, Nadia M. et al. [34] proposed permutation Renyi entropy (PEr), and it was successfully applied to analyze the changes in childhood epilepsy EEG signals. Soon multiscale permutation Renyi entropy (MPEr) [12] was proposed. In order to fully consider the multi-channel characteristics of EEG signals, some researchers used multivariate algorithms and PCA dimensionality reduction [25,26]. Obviously, these studies lay a good foundation for the applications of EEG signals.

Motivated by the above discussions, in this paper, we proposed a modified multivariate PE measure method to analyze EEG signals with 21 channels, where PCA is employed to shrink the dimension of the data. In fact, we try to improve the method based on the Bandt–Pompe ordinal patterns [26] from the multiple time series. Specifically, we use the Bandt–Pompe ordinal patterns of corresponding positions to build new patters and to increase the number of patterns for better performance. For the one hand, we provide a new method for the multivariable time series, for the another hand, EEG signals before mental arithmetic task and during the mental arithmetic task are analyzed.

The rest of this paper is divided into the following parts. Section 2 introduces the basic principles of PCA and permutation entropy, then gives the derivation process of two multivariate permutation entropy in detail. Section 3 illustrates the complexity analysis of chaotic systems. We apply the new algorithm to analyze the EEG signals and make some comparisons in Section 4. Finally, we summarize this article and indicate the future work.

## 2. Complexity Measure Methods

### 2.1. PCA and Normalization of the Multivariable Time Series

#### 2.1.1. PCA

In nature, there are many signals that require multi-channel data to be relatively accurately described (for example, the EEG signals). The selection of signal channels becomes a problem to solve. Some are solved by designing the channel selection algorithm, while some are solved by fusing different channels to retain the main information and remove the redundant information, so as to achieve the purpose of dimensionality reduction. The dimensionality reduction method is divided into linear and non-linear dimensionality reduction. Here, we mainly introduce the PCA method in linear dimensionality reduction. The steps of PCA is given as follows [35,36].

Step 1: Suppose that there is a multivariable time series $\left\{x_{i}={\left[x_{i,1},x_{i,2},\cdots ,x_{i,k}\right]}^{T}\right\}$ with k dimensional, where i=1,2,⋯,n, and *n* is the length of time series. Let n>k, construct sample matrix, where the following normalized transformation is applied

(1) $$Z_{i,j}=\frac{x_{i,j}-\bar{x_{:,j}}}{s_{j}},$$

where $\bar{x_{:,j}}=\frac{\sum_{i=1}^{n}x_{i,j}}{n}$ and $s_{j}=\sqrt{\frac{\sum_{j=1}^{n}{\left(x_{i,j}-\bar{x_{:,j}}\right)}^{2}}{n-1}}$. In this paper, $x_{:,j}≅\left\{x_{i,j},i=1,2,\cdots \right\}$ and $x_{i,:}≅\left\{x_{i,j},j=1,2,\cdots \right\}$.

Step 2: Calculate the correlation coefficient matrix of matrix Z, and it is denoted as

(2) $$R=\frac{Z^{T}Z}{n-1}.$$

Step 3: Solve the characteristic equation of matrix R, which is $\left|R-\lambda I_{k}\right|=0$. Thus, we can get a series of characteristic roots $\left\{\lambda_{j}:j=1,2,\cdots ,k\right\}$. Then the shrinked dimension *m* is estimated by ∑j=1mλj∑j=1mλj∑j=1kλj∑j=1kλj≥0.85. It makes the utilization rate of information reach more than 85%.

Step 4: For each $\lambda_{j}\left(j=1,2,\cdots ,k\right)$, solve the equations $\mathrm{Rb}=\lambda_{j}b$, we can obtain the unit eigenvector $b_{j}^{o}$.

Step 5: Transfer the standardized index variable conversion to the main component, and it is

(3) $$U_{i,j}=z_{i}^{T}b_{j}^{o}$$

where j=1,2,⋯,m, and $z_{i}$ is the *i*th vector in the matrix Z. Thus, we get a *m*-dimensional time series with length *n*.

#### 2.1.2. Normalization

Suppose that the original time series is defined by

(4) $$X=\left[\begin{array}{cccc} x_{1,1} & x_{1,2} & \cdots & x_{1,n}\\ x_{2,1} & x_{2,1} & \cdots & x_{2,n}\\ \vdots & \vdots & \ddots & \vdots \\ x_{k,1} & x_{k,2} & \cdots & x_{k,n} \end{array}\right],$$

where *n* is the length of sequence and *k* is the dimension of the time series. To obtain the normalization of the multivariable time series, there are three steps.

Step 1: Normalize the original time series, and it is given by

(5) $${\tilde{x}}_{i,j}=\frac{\max\left(x_{i,:}\right)-x_{i,j}}{\max\left(x_{i,:}\right)-\min\left(x_{i,:}\right)},$$

where i=1,2,⋯,k, j=1,2,⋯,n, and $x_{i,:}$ represents the *i*th line of the time series.

Step 2: PCA of the time series ${\tilde{x}}_{i,j}$, and shrinks the dimension of the time series. The new *m*-dimensional time series is denoted as

(6) $$Y=\left[\begin{array}{cccc} y_{1,1} & {}_{1,2} & \cdots & y_{1,n}\\ y_{2,1} & y_{2,2} & \cdots & y_{2,n}\\ \vdots & \vdots & \ddots & \vdots \\ y_{m,1} & y_{m,2} & \cdots & y_{m,n} \end{array}\right],$$

where m<k.

Step 3: Normalize the *m*-dimensional time series, and it is given by

(7) $${\tilde{y}}_{ij}=\frac{\max\left(y_{i,:}\right)-y_{i,j}}{\max\left(y_{i,:}\right)-\min\left(y_{i,:}\right)}$$

where i=1,2,⋯,m; j=1,2,⋯,n, and $y_{i,:}$ represents the *i*th line of the time series. Thus, the obtained time series $Y=\left[\begin{array}{cccc} {\tilde{y}}_{1,1} & {\tilde{y}}_{1,2} & \cdots & {\tilde{y}}_{1,n}\\ {\tilde{y}}_{2,1} & {\tilde{y}}_{2,2} & \cdots & {\tilde{y}}_{2,n}\\ \vdots & \vdots & \ddots & \vdots \\ {\tilde{y}}_{m,1} & {\tilde{y}}_{m,2} & \cdots & {\tilde{y}}_{m,n} \end{array}\right]$ can be used to measure the complexity.

It should be noted that, if the PCA process is not employed to the multi-dimensional time series *X*, only Step 1 is necessary for the normalization. In the coming sections, the complexity algorithm is designed based on the normalized time series.

### 2.2. Multivariable Ordinal Pattern Representations

**Figure 1:**
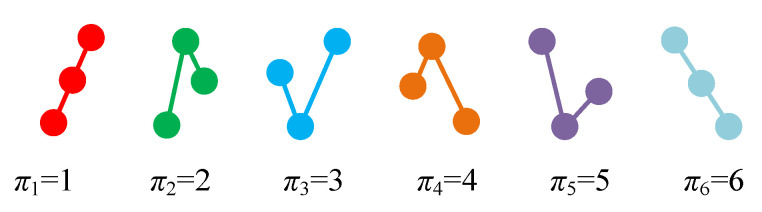
The six patterns for *d* = 3.

Bandt and Pompe [17] proposed the ordinal patterns to detect the complex patterns in the nonlinear time series. For a given time series $\{x_{n}:n$ = 1, 2, 3, ⋯, N} and a given parameter *d*, the reconstructed vectors are defined by

(8) $$\Phi_{i}=\left\{x_{i},x_{i+1},\cdots ,x_{i+d-1}\right\},$$

where *i* = 1, 2, ⋯, N−d+1. For each vector $X_{i}$, it is resorted by ascending sort as

(9) $$x_{i+r_{0}}\leq x_{i+r_{1}}\leq \cdots \leq x_{i+r_{d-1}}$$

where π = $\left(r_{0},r_{1},\cdots ,r_{d-1}\right)$ are the index of the vector $\Phi_{i}$. Obviously, there are d! possible π. If *d* = 3, there are six ordinal patterns, namely, $\left\{\pi_{1},x_{1}\leq x_{2}\leq x_{3}\right\}$, $\left\{\pi_{2},x_{1}\leq x_{3}\leq x_{2}\right\}$, $\left\{\pi_{3},x_{2}\leq x_{1}\leq x_{3}\right\}$, $\left\{\pi_{4},x_{3}\leq x_{1}\leq x_{2}\right\}$, $\left\{\pi_{5},x_{2}\leq x_{3}\leq x_{1}\right\}$, $\left\{\pi_{6},x_{3}\leq x_{2}\leq x_{1}\right\}$. The six patterns are presented in Figure 1. Let $\pi_{\theta }$ = θ(θ = 1, 2, ⋯, d!). If $X_{i}$ is of pattern $\pi_{\theta }$ and $s_{i}=\theta$, then we can get a symbol time series $\{s_{i},i$ = 1, 2, ⋯, N−d+1}.

**Remark** **1.** *For convenience, we use symbols to represent the obtained patterns. Thus, a pattern time series is introduced.*

Suppose that there is a multivariable time series after normalization, and it is defined by

(10) $$\left\{x_{i,j}:i=1,2,\cdots ,m;j=1,2,\cdots ,n\right\}=\left[\begin{array}{cccc} x_{1,1} & x_{1,2} & \cdots & x_{1,n}\\ x_{2,1} & x_{2,2} & \cdots & x_{2,n}\\ \vdots & \vdots & \ddots & \vdots \\ x_{m,1} & x_{m,1} & \cdots & x_{m,n} \end{array}\right],$$

where *m* is the dimension, and *n* is the length of the time series.

By using the Bandt–Pompe pattern for each time series $\left\{x_{i,j}:j=1,2,\ldots ,n\right\}$, we can get pattern series for each time series $x_{i,:}$, and define the pattern series by

(11) $$\left\{s_{i,j}:i=1,2,\cdots ,m,j=1,2,\cdots ,n-d+1\right\}$$

Above pattern series is obtained based on the principle of the original Bandt–Pompe method. Recently, Mohr M. et al. [26] improved the patterns for multi variable time series. This method is to capture new patterns based on the obtained patterns. The new pattern vector is given as

(12) $$g_{j}=\left[s_{1,j},s_{2,j},\cdots ,s_{m,j}\right].$$

In simulations, we need to change the patters to symbols or numbers. Here, it is defined by

(13) $$\varphi_{j}=s_{1,j}\times d{!}^{m-1}+s_{2,j}\times d{!}^{m-2}+\cdots +s_{m,j}\times d{!}^{0}.$$

As a result, a symbol time series related to the patterns $\{\varphi_{i},i$ = 1, 2, ⋯, N−d+1} is obtained for entropy estimation.

**Figure 2:**
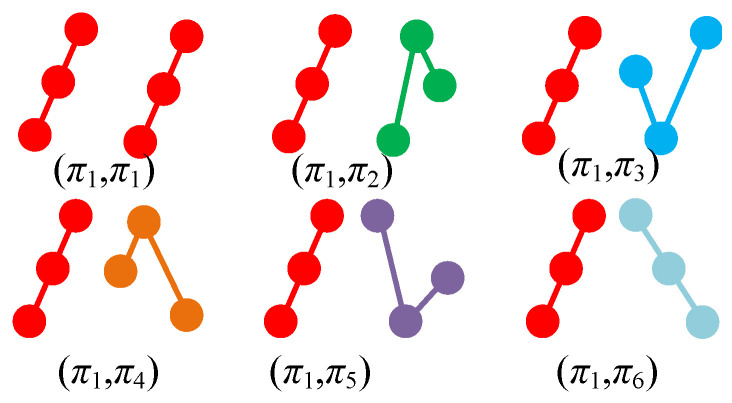
The six possible cases for $d=3(\pi_{1})$ and variable-dimension *m* = 2.

Here, we consider the combination of obtained multivariate ordinal patterns, which means that there could be $d{!}^{m}$ possible patterns from the vectors. Specifically since each *s* has d! possiblities and thus there are $d{!}^{m}$ patterns in the new symbol time series. Let m=2,d=3, as shown in Figure 2, an example is presented to show the details of the new patterns and the case for $d=3(s_{1,i}=\pi_{1})$. Thus, there are six possible patterns for each pair of symbols $\left(s_{1,j},s_{2,j}\right)$, and there are $6^{2}=36$ possible patterns for the final results.

For the modified multivariable PE (MPE) compelxity measure method, the vectors $X_{i}$ is defined by

(14) $$X_{i}=\left\{x_{1,i},x_{2,i},\cdots ,x_{m,i}\right\},$$

to build the pattern series, where *i* = 1, 2, ⋯, *n*. According to the Bandt–Pompe patterns, we have a symbol time series $\{s_{i}:i$ = 1, 2, ⋯, N}.

### 2.3. Multivariable Permutation Entropy Algorithms

The PE algorithm [17] is calculated based on $\{s_{i}:i$ = 1, 2, ⋯, N−d+1}, and it is defined by

(15) $$p\left(\pi_{\theta }\right)=\frac{\#\left\{s_{i}\left|i\leq N-d+1;s_{i}=\theta \right.\right\}}{N-d+1},$$

where symbol # represents the number, and θ=1,2,⋯,d!. According to the Shannon entropy definition, the PE algorithm is denoted as

(16) $$\mathrm{PE}\left(x,d\right)=-\frac{1}{S_{\max}}\sum_{\theta =1}^{d!}p\left(\pi_{\theta }\right)\ln p\left(\pi_{\theta }\right),$$

where $S_{max}=S\left[P_{e}\right]=ln\left(d!\right)$.

The multivariable PE (MPE) algorithm is also defined based on the pattern series. For the obtained symbol series $\{s_{i}:i$ = 1, 2, ⋯,N}, the corresponding probability distribution is denoted as

(17) $$p\left(\pi_{\theta }\right)=\frac{1}{N}\#\left\{s_{i}\left|i\leq N;s_{i}=\theta \right.\right\},$$

where symbol # represent number. According to the Shannon entropy definition, MPE is denoted as

(18) $$\mathrm{MPE}\left(x,d\right)=-\frac{1}{S_{\max}}\sum_{\theta =1}^{d!}p\left(\pi_{\theta }\right)\ln p\left(\pi_{\theta }\right),$$

where θ=1,2,⋯,d!, and $S_{max}=S\left[P_{e}\right]=ln\left(d!\right)$.

In this paper, based on the symbol time series $\{\varphi_{i},i$ = 1, 2, ⋯, N−d+1}, a complexity measure method for multivariable time series is proposed. Obviously, it is a modified multivariable complexity measure algorithm, and we call it modified MPE (MMPE) algorithm. For the symbol time series from the multivariable time series, and its probability distribution is defined by

(19) $$p\left(\pi_{\theta }\right)=\frac{\#\left\{\varphi_{i}\left|i\leq N-d+1;\varphi_{i}=\theta \right.\right\}}{N-d+1},$$

where symbol # represents number. According to the Shannon definition, the MMPE is denoted as

(20) $$\mathrm{MMPE}\left(x,d\right)=-\frac{1}{S_{\max}}\sum_{\theta =1}^{d!}p\left(\pi_{\theta }\right)\ln p\left(\pi_{\theta }\right).$$

Here, $\theta =1,2,\cdots ,d{!}^{m}$, and $S_{max}=S\left[P_{e}\right]=ln\left(d{!}^{m}\right)$.

The three algorithms mentioned above are all designed based on the Shannon entropy, but the pattern series is obtained through different methods. The PE algorithm measures complexity of a single time series, while MPE and MMPE estimate complexity of multiple time series. The embedded dimension of MPE is the dimension of the phase space or the dimension of the multiple time series, so it is fixed. However, the embedded dimension of MMPE can be adjusted for better estimation results.

### 2.4. Discussion of the Complexity Measurement Methods

Firstly, the characteristics of the proposed MMPE algorithm are summarized, and we discussed how to choose proper algorithm.

(1) The new approach can have more patterns compared with the existing methods like PE and MPE algorithms, and there are $d{!}^{m}$ patterns in the new approach for multivariate time series, where *d* is the embedding dimension and *m* is the dimension of the time series.
(2) Generally, if *d* takes larger values, there are more patterns. In the real applications, the embedded dimension *d* can be {2, 3, 4, 5}.
(3) When *m* becomes to be larger, the number of patterns increase significantly. In the real applications, *m* could be a large value. Thus, we need a method to shrink the dimension. In this paper, the PCA algorithm is employed to decrease the dimension of the multivariable time series. In the real application, we suggest that the value of *m* could be smaller than 5.
(4) In general, more patterns mean better recognition of nonlinearity in the time series. Two reasons are presented. Firstly, less patterns mean less computation, but it losses more information. Secondly, if there are more items, the obtained patterns contain more information regarding the nonlinearity in the time series.
(5) In simulations, it is found that there are some “missing” Bandt–Pompe ordinal patterns for some chaotic systems. In fact, the chaotic time series are not random time series. So there is always some “missing” ordinal patterns for the chaotic time series and other nonlinear time series if they are not totally random.
(6) MMPE is an improved version of PE algorithm and MPE algorithm. Its time complexity is $O_{n}$.

Secondly, until now, there is no uniform definition for the concept of complexity. Scientists define the complexity in their own fields. For instance, in year 1995, Horgan [37] pointed out that there are more than 45 different kinds of complexity definitions such as information, entropy, time complexity, space complexity, semantic complexity and Kolmogorov complexity. As a result, lots of complexity measure methods are proposed for nonlinear time series. Those methods have different characteristics. Moreover, we summarize the advantages or disadvantages of several widely used complexity measure methods, and the results are illustrated in Table 1. Those methods are designed for the one dimensional nonlinear time series.

Thirdly, at present, except MPE, there are also many other multivariable complexity measure algorithms. Overall, these methods are designed based on the existing complexity measure methods which contain phase-space reconstruction. For instance, PE algorithm has phase-space reconstruction for the one dimensional time series for patterns, so it can be modified for complexity measure of multivariable time series. MMPE is different from those multivariable complexity measure algorithms, although it is also designed based on the phase-space reconstruction. This method gets its patterns based on the Bandt–Pompe patterns of each time series. Thus, we can estimate complexity using more information. Meanwhile, we introduced the PCA method to shrink the dimension of the multiple time series and to keep more information for the whole system.

Fourthly, it is still a challenge to analyze complexity of nonlinear time series accurately. Meanwhile, those entropy methods and complexity methods cannot be used to detect the existence of chaos in the nonlinear systems. There are also some methods which can be used to detect nonlinearity in the time series. For instance, the ANNs has been implemented to predict the chaos in larger horizons [41]. Moreover, it is found that the physiological time series can be chaotic time series since they have positive Lyapunov exponents. Specifically, Yang et al. [42] investigated the chaotic feature of EEG signal based the Poincaré surface.

## 3. Complexity Analysis of Chaotic Systems

**Figure 3:**
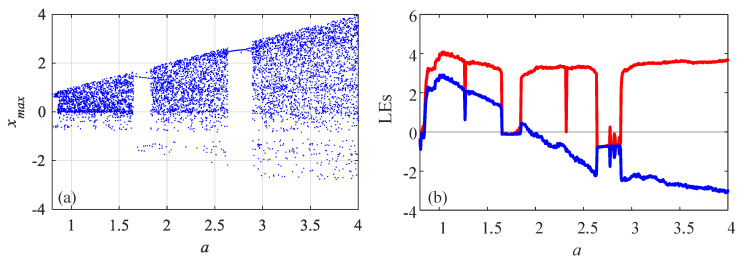
Dynamical analysis results of the 2D-SIMM system with the variation of parameter *a*. (**a**) Bifurcation diagram; (**b**) Lyapunov exponents.

Firstly, complexity of 2D-SIMM chaotic map [43] is analyzed. The system is defined by

(21) $$\left\{\begin{array}{c} x_{i}=a\sin\left(\omega y_{{}^{i-1}}\right)\sin\left(\frac{b}{x_{i-1}}\right)\\ y_{i}=a\sin\left(\omega x_{i}\right)\sin\left(\frac{b}{y_{i-1}}\right) \end{array}\right.,$$

where *a*, *b* and ω are the system parameters, and $a,b,\omega \in \left(0,+\infty \right)$. Let ω=π,b=3, and *a* varies from 0.4 to 4 with step size of 0.0064. The bifurcation and Lyapunov exponents (LEs) of the system are plotted in Figure 3. It shows that the system has rich dynamics with the variation of parameter *a*. Specifically, there are periodic windows and chaotic intervals with the increase of parameter *a*. The length of time series for complexity measure about this system is 10,000.

**Figure 4:**
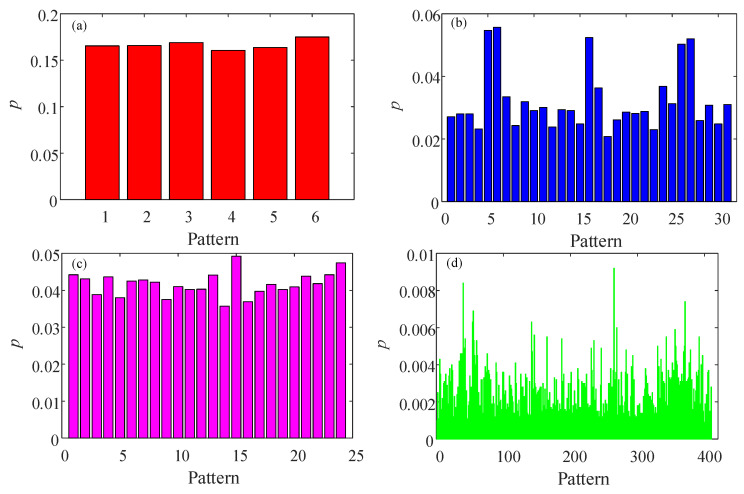
Probability distribution of the system based on different algorithms and *d*. (**a**) *d* = 3 and PE algorithm; (**b**) *d* = 3 and MMPE algorithm; (**c**) *d* = 4 and PE algorithm; (**d**) *d* = 4 and MMPE algorithm.

**Figure 5:**
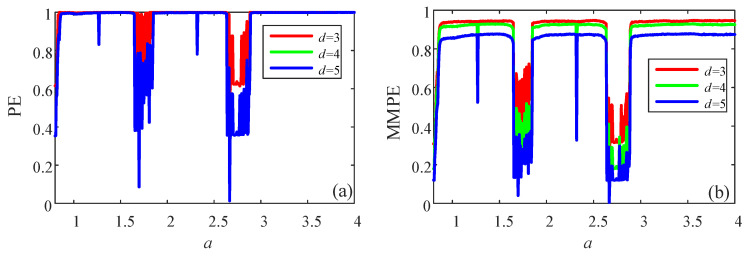
Complexity measure results of the 2D-SIMM system with the variation of parameter *a* and different algorithms. (**a**) PE algorithm; (**b**) MMPE algorithm.

Firstly, fix ω=π,a=1,b=3, and the initial condition as $x_{0}$ = 0.45, $y_{0}$ = 0.95, a segment of time series is obtained to test different algorithms. When *d* = 3, the probability distribution of PE algorithm is shown in Figure 4a, while the probability distribution of MMPE algorithm is given in Figure 4b. It shows that there are more patterns in the MMPE algorithm compared with PE algorithm. Moreover, when *d* = 4, the probability distributions of PE and MMPE are illustrated in Figure 4c,d. As shown in Figure 4d, there are about 400 recognized patterns. In fact, there are 576 possible patterns. Thus, compared with PE algorithm, MMPE algorithm can estimate complexity using more patterns and it should have better analysis results. As shown in Figure 5, compared with the complexity analysis of PE, analysis results of MMPE algorithm have better performance with the parameter *a* and better degree of differentiation with different embedded dimension *d*. In conclusion, MMPE has better performance for complexity measure of the discrete chaotic system.

Sun et al. [44] simplified the Lorenz system, and it is defined as

(22) $$\left\{\begin{array}{c} \dot{x}=10\left(y-x\right)\\ \dot{y}=-xz+\left(24-4c\right)x+cy\\ \dot{z}=xy-\frac{8}{3}z \end{array}\right.,$$

where *c* is the bifurcation parameter. When c∈(−1.59,7.75), the system is generally chaotic. Moreover, the length of time series for complexity measure about this system is 20,000.

**Figure 6:**
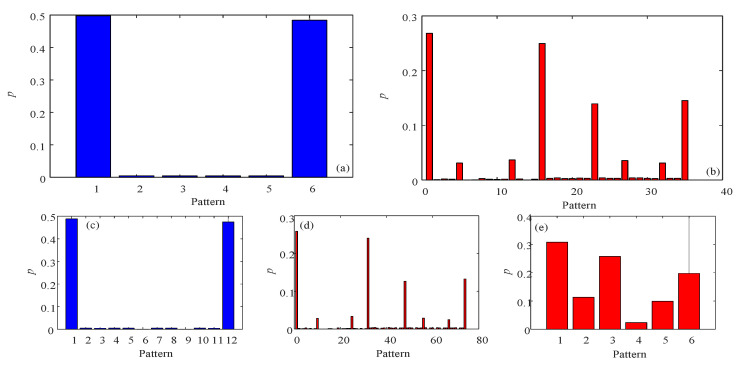
Probability distribution of the simplified Lorenz system based on different algorithms and *d*. (**a**) *d* = 3 and PE algorithm; (**b**) *d* = 3 and MMPE algorithm; (**c**) *d* = 4 and PE algorithm; (**d**) *d* = 4 and MMPE algorithm; (**e**) *d* = 3 and MPE algorithm.

**Figure 7:**
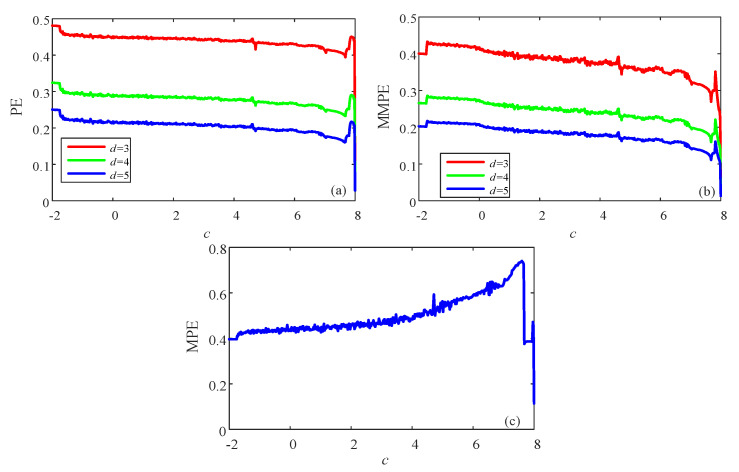
Complexity measure results of the simplified Lorenz system with the variation of parameter *a* and different algorithms. (**a**) PE algorithm; (**b**) MMPE algorithm; (**c**) MPE algorithm.

Complexity analysis results using PE algorithm, MPE algorithm and MMPE algorithm are presented. Firstly, the probability distribution of different algorithms and embedded dimension *d* are shown in Figure 6. For the MPE algorithm, its embedded dimension *d* is fixed, which is the dimension of the system. For system (21), it is 3. There could be six possible patterns, and the probability distribution of the 6 patterns is shown in Figure 6e. When *d* = 3, compared with PE algorithm, MPE algorithm and MMPE algorithm show more information regarding the nonlinearity of the time series. When *d* = 4, PE and MMPE show more patterns, but the number of patterns increases remarkably in MMPE algorithm. Meanwhile, the complexity analysis results are presented in Figure 7. It shows that the PE algorithm and MMPE algorithm can measure complexity of the continuous chaotic system effectively, and the trend agrees better with the corresponding Lyapunov exponents. In Figure 7c the MPE algorithm does not show a satisfying result when comparing with the results form the largest Lyapunov exponents. It shows that the MMPE algorithm are effective for the complexity measurement of the chaotic systems.

**Figure 8:**
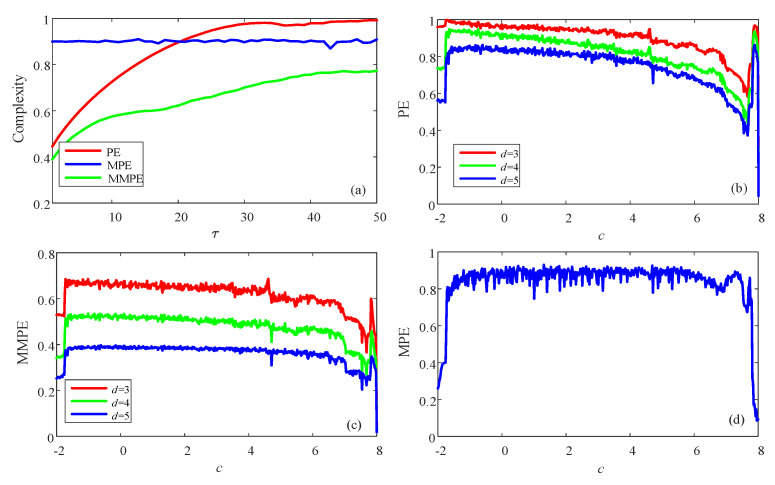
Complexity of the simplified Lorenz system with different sample periodic τ. (**a**) Complexity with different sample periodic τ; (**b**) PE algorithm and τ=25; (**c**) MMPE algorithm and τ=25; (**d**) MPE algorithm and τ=25.

For the continuous chaotic systems, it is important to choose a proper time step for the simulation and then for the complexity measurement. It should be noted out that the simplified Lorenz system is solved by using the fourth-order Runge–Kutta method with h=0.01. If we want to obtain time series with larger time step, we can sample the obtained time series. It shows that the step size is a key factor for dynamics of the time series from the continuous chaotic systems [45]. Here, for the obtained time series, different τ is used to sample the data. Since the time series are discrete sequence, the value of τ in this paper is integer numbers. Complexity results versus the sample periodic τ is presented in Figure 8a. It shows that MPE analysis results do not increase with the sample periodic τ, while both PE and MMPE analysis results increase with the sample periodic τ. However, PE analysis results reach one while MMPE reach a certain value. Moreover, let τ=25, complexity of the simplified Lorenz system with *c* is analyzed, and the results obtained by PE, the MMPE and MPE as shown in Figure 8b–d, respectively. It shows that more satisfying results are obtained compared the time series with original time series (τ=1). However, results from the MMPE algorithm and PE algorithm match better with the corresponding Lyapunov exponents.

According to the analysis above, the proposed MMPE algorithm can extract more patterns comparing with the existing methods. Thus, in this paper, we will use it to analyze the complexity of EEG data of different states.

## 4. Determine State of EEG Signals

### 4.1. Data Description

**Figure 9:**
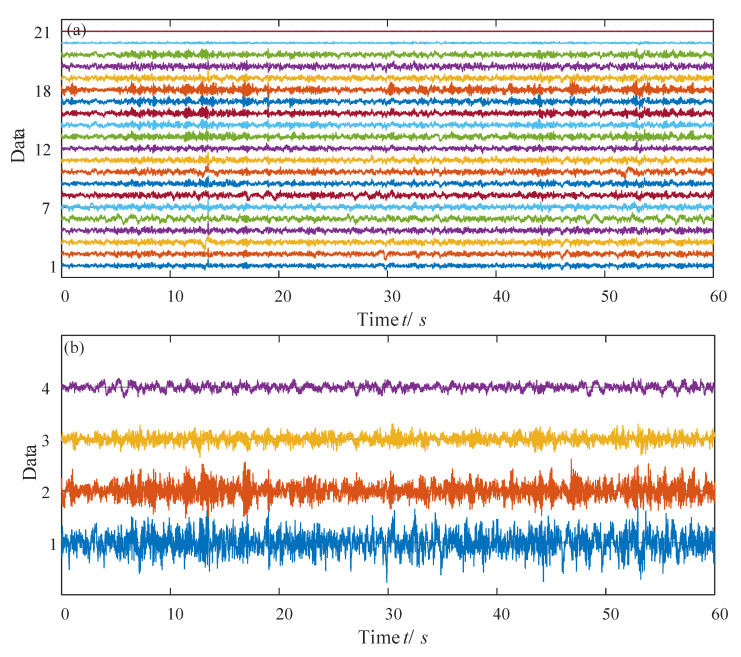
Sample data from Subject02_2 of 60 s. (**a**) All the data; (**b**) PCA results with dimension four.

The EEG signal database used in this article is a public database provided by physionet. The website is https://www.physionet.org/content/eegmat/1.0.0/ (17 December 2018. Version: 1.0.0). This database was contributed by Igor Zyma, Sergii Tukaev, and Ivan Seleznov, National Technical University of Ukraine “Igor Sikorsky Kyiv Polytechnic Institute”, Department of Electronic Engineering [46,47]. The EEGs were recorded monopolarly using Neurocom EEG 23-channel system (Ukraine, XAI-MEDICA). The silver/silver chloride electrodes were placed on the scalp. According to the International 10/20 scheme, all electrodes are referenced to the interconnected ear reference electrode. The database contains EEG records of subjects before and during the execution of mental arithmetic tasks. All signals are filtered with a high-pass filter with a cut-off frequency of 30 Hz and a power line notch filter (50 Hz). Then the artifacts are removed by independent component analysis, which is a clean signal that can be directly used for analysis. The database has a total of 36 samples. The sample age is 16–26 years old. Each sample collected two segments of signals, and recorded their EEG data when they were calm and the EEG data when they performed simple arithmetic tasks. The lengths are, respectively, three minutes and one minute. The simple arithmetic task is the subtraction of two numbers. We use all 36 samples to get a valid systematic conclusion. There are 21 channels of data for each subject. As an example, time series of Subject02 during mental arithmetic task are presented in Figure 9, where Figure 9a shows the original time series and Figure 9b illustrates the PCA results with dimension four.

### 4.2. Complexity Analysis

#### 4.2.1. MMPE Analysis

The data set has 31,000 points of data for the 60 s. Here, we divide the data into some windows with the length 10,000. The distance of two neighbor windows is to check their index of the first data and the distance is 500. For instance, the first window contains the data points with index 1~10,000, while the second window contains the data points with index 501~10,500. As a result, there are 40 measuring results of each subject for the two states. As for the transient behaviors in the used EEG data, it has already been handled in the data base. Moreover, we use the mean values as the final results, and use the box plots to illustrate the difference.

**Figure 10:**
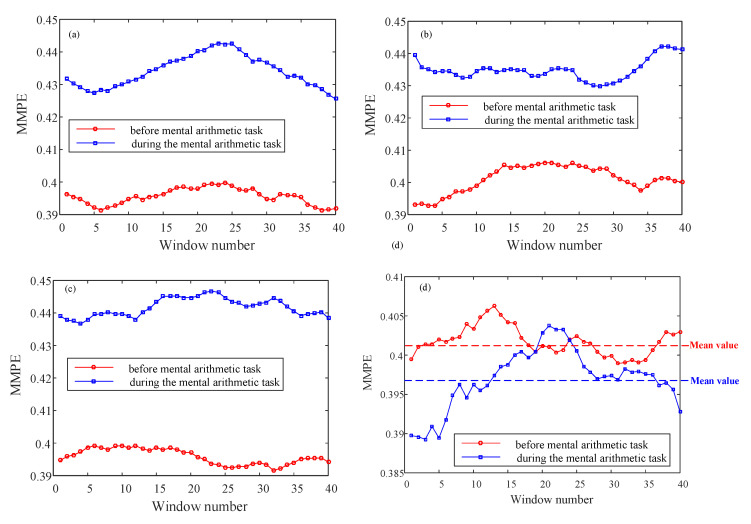
Complexity measure results of different subject with different windows. (**a**) Subject01; (**b**) Subject16; (**c**) Subject30; (**d**) Subject36.

Figure 10 illustrates that the analysis results for the Subject01, Subject16, Subject30 and Subject36. It shows that MMPE can distinguish the two states, where the dimension of data after PCA is four. It shows that the subjects during mental arithmetic task have higher complexity comparing with the state before mental arithmetic task.

To further confirm the analysis results as shown in Figure 10, we calculate the MMPE complexity of all the 36 subjects. As mentioned above, the complexity is measured under different windows. For each subject, we have 40 measure values for each state and the mean value is used for the statistic analysis.

**Figure 11:**
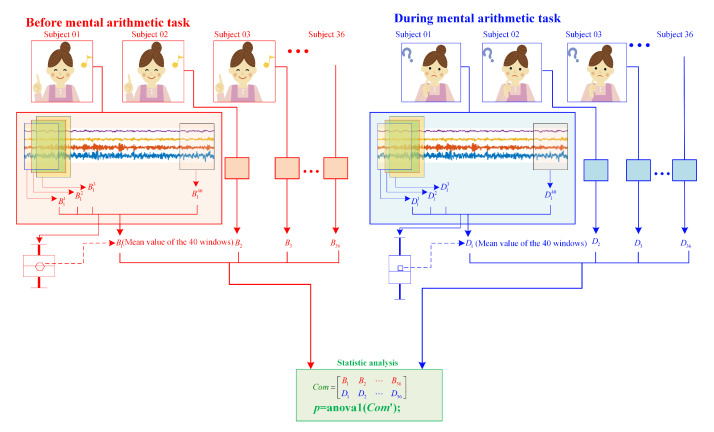
The steps of complexity analysis of EEG signals. Here, we suppose that the complexity of subjects before mental arithmetic tasks represented by $B_{1∼36}$, while complexity of subjects during mental arithmetic tasks represented by $D_{1∼36}$.

Firstly, statistic analysis of the three cases with *d* = 3, 4 and 5 are carried out, where the mean values are used to represent the complexity of each subject for different states. The whole process for complexity analysis and its statistic analysis of EEG signals can be found in Figure 11. For a given *d* and the obtained time series, the following steps are applied.

Step 1: We checked the distribution of the MMPE values. It shows that the two segments of data ($Seq_{B}=\left[B_{1},B_{2},\cdots ,B_{36}\right]$ and $Seq_{D}=\left[D_{1},D_{2},\cdots ,D_{36}\right]$) obey the normal distribution. As a result, $Seq_{B}$ is the MMPE values drawn from the subjects before mental arithmetic task, and $Seq_{D}$ is the MMPE values drawn from the subjects during mental arithmetic task.

Step 2: The two independent test data simulated based on the EEG signals, namely, $Seq_{B}$ and $Seq_{D}$, are used to do the statistical analysis. Thus, the factor is the ‘Group’, group $Seq_{B}$ and group $Seq_{D}$. They are measured from same subjects but two different states.

Step 3: Here, the null hypothesis means that subjects during mental arithmetic task and before mental arithmetic task have the same MMPE complexity as they are during mental arithmetic task.

Step 4: We use the Matlab function “[p,anovatab,stats]=anova1(x,group)” to do the statistic test. We take p=0.05 as the level of statistical significance for the tests.

**Figure 12:**
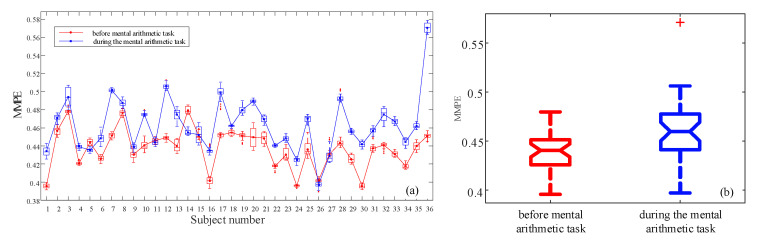
MMPE measure results with *d* = 4. (**a**) Analysis results for each subject using boxplot; (**b**) Boxplot of the two states.

**Figure 13:**
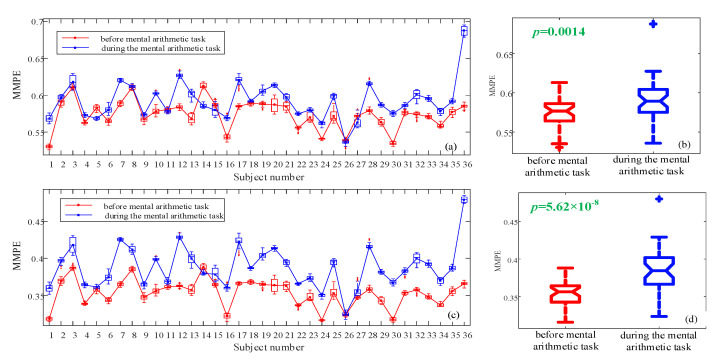
MMPE measure results with different *d*. (**a**) *d* = 3, analysis results for each subject using boxplot; (**b**) *d* = 3, boxplot of the two states; (**c**) *d* = 5, analysis results for each subject using boxplot; (**d**) *d* = 5, boxplot of the two states.

Secondly, the measure results are plotted using box plots for each subject, and the results are shown in Figure 12a and Figure 13a,c. Obviously, for very few subjects, MMPE shows higher complexity for those before mental arithmetic tasks. Most of the subjects show higher MMPE complexity during mental arithmetic tasks, and we should consider the sample differences and the data validation. Moreover, as shown in those figures, when *d* takes larger values, the analysis results are more accurate due to that there are more patterns are identified.

The detail information for *d* = 4 is illustrated in Table 2. It shows that the *p*-value is 0.001. Since it is smaller than 0.05, we could reject the null hypothesis. It means that the MMPE complexity measure results can identify the two states, and the state during mental arithmetic tasks has higher complexity. Meanwhile, the rank of *p*-values is ‘(*p*-value = 0.0014) d=3’ > ‘(*p*-value = 0.001), *d* = 4’ > ‘(*p*-value = 5.62 × $10^{-8}$), *d* = 5’. Obviously, MMPE algorithm with *d* = 5 shows the best analysis results, and it shows the effectiveness of the proposed MMPE algorithm.

#### 4.2.2. MPE Analysis

**Figure 14:**
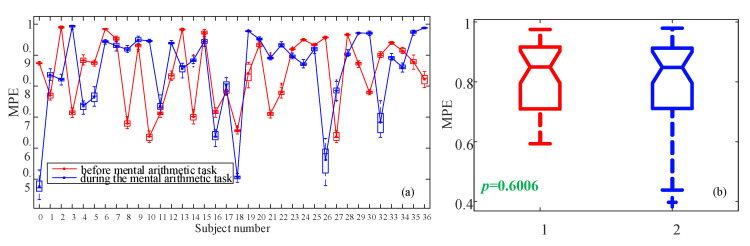
MPE analysis results. (**a**) Analysis results for each subject using boxplots; (**b**) Boxplot of the two states.

In Section 2, we introduced an MPE algorithm. Here we use the MPE algorithm to measure the complexity of the EEG data after PCA, where the dimension is four. The analysis results are shown in Figure 14. It shows that MPE cannot identify the two states. Furthermore, *p*-value is 0.6006 which is larger than 0.005. Thus, it means the two states cannot be distinguished statistically.

Furthermore, as mentioned above, Figure 12a, Figure 13a,c and Figure 14a are the complexity analysis results of all subjects, and the results are presented as box plots. Thus, we have distinguished the state before mental arithmetic task and the state during the mental arithmetic task EEG states from the population in general. Meanwhile, the Matlab anova1(·) function documentation clearly states that its inputs are assumed to be independent. Since there are two kinds of box plots, to avoid the possible misunderstanding, two remarks are presented. In our experience, the inputs of anova1(·) function are two independent states.

**Remark** **2.** *Figure 12a, Figure 13a,c and Figure 14a are the complexity analysis results of all subjects, and the results are presented in box plots since the complexity of each subject are calculated using the sliding windows.*

**Remark** **3.** *Figure 12b, Figure 13b,d and Figure 14b are plotted based on the mean values of the 40 windows in each subject under the two states. Thus, the input of function anova1(·) can be divided into two parts. One is a sequence contains the mean values of subjects before mental arithmetic task. The other is a sequence contains the mean values of subjects during the mental arithmetic task. It means that the inputs include two independent parts.*

#### 4.2.3. The Necessity of PCA

**Figure 15:**
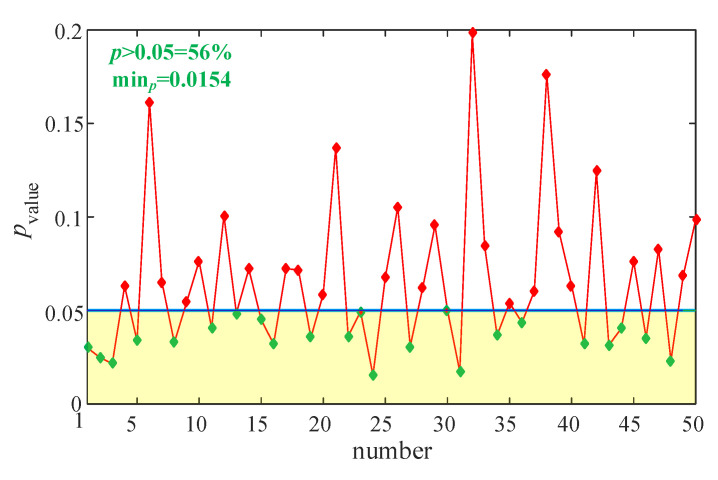
MMPE measure results using randomly chosen data where *m* = 4 and *d* = 4.

Here, we investigate the fact that whether PCA is necessary for the MMPE algorithm. We randomly choose four channels to estimate MMPE complexity and calculate the *p*-values. The experiments are run 50 times and the results are illustrated in Figure 15. When *p*-value is smaller than 0.05, the four channels chosen can be used to estimate MMPE complexity and identify the two states. It shows in Figure 15 that the minimum *p*-value is 0.0154, and it is larger than the PCA based analysis. Obviously, the rate for *p*-value larger than 0.05 is 56%. Thus, we can see that the PCA is necessary for the MMPE algorithm when the dimension of the system is high or the number of time series is large.

According to the experiment results, MMPE is effective for complexity analysis of multiple time series. In addition, PCA is necessary to shrink the dimension of the phase space since it can extract necessary information from the phase space. Moreover, it shows that MPE algorithm cannot be used to analyze complexity of the EEG signals, although it has the similar effect for the complexity analysis of chaotic systems.

## 5. Conclusions

In this paper, in order to measure the complexity of multi-dimensional time series, we proposed a modified multivariate permutation entropy. The analysis results showed that the proposed MMPE algorithm can extract more patterns comparing with the existing methods. The analysis of discrete chaotic system shows that, compared with the complexity analysis of PE, the analysis results of MMPE algorithm have better performance with the parameter *a* and better degree of differentiation with different embedded dimension *d*. Meanwhile, the simulation analysis results prove that MMPE is effective for the complexity measurement of continuous chaotic systems.

As an application, MMPE was applied to analyze the complexity of EEG including the person before mental arithmetic task and during mental arithmetic task. In order to decrease the dimensionality of the signals, we firstly reduced the dimensionality of the EEG signals by PCA. The measure results show that participants during mental arithmetic task have higher MMPE complexity. Moreover, when *d* takes larger values, the analysis results are better due to that more patterns are identified. Then a statistical analysis was performed on all the samples, and the results showed that the larger *d*, the better the two different states are distinguished. According to the analysis of anoval and boxplot, MMPE can effectively distinguish the two states. As a comparison, we performed the same analysis with MPE, and the results showed that it could not distinguish the two states. Finally, it was proved that PCA is necessary by comparing with the calculation results of the complexity of randomly selected channel data. Our next work will focus on the analysis of EEG signals of different types of diseases, and find a more effective way which combines the neural networks to make more precise distinctions.

## Figures and Tables

**Table 1:** Comparison of several complexity measure methods.

Method	Characteristic	Advantages	Disadvantages
ApEn [13] SampEn [14] FuzzyEn [15]	Time domain, Phase-space reconstruction Distance between the vectors.	Short time series.	$O_{n^{2}}$, slow, Not for time series with long length
PE [17]	Time domain, Patters from vectors, Shannon entropy.	$O_{n}$, Fast.	It cannot detect the periodic state some times, Limited by the patters.
Dispersion entropy [16]	Distribution, Patters, Shannon entropy	$O_{n}$, fast, Improved version of PE	−
Intensive statistical complexity measure [38]	It combines PE algorithm and the probability distribution	$O_{n}$, fast, Improved version of PE	Similar as PE algorithm
C_0_ [39]	Frequency domain	FFT, Fast	−
Spectral entropy [40]	Frequency domain	Fast; FFT Shannon entropy	−

**Table 2:** ANOVA analysis.

Source	SS	df	MS	F	Prob > F
Columns	0.01202	1	0.01202	16.91	0.001
Error	0.04978	70	0.00071		
Total	0.0618	71			

## Data Availability

Publicly available datasets were analyzed in this study. This data can be found here: https://www.physionet.org/content/eegmat/1.0.0/ (17 December 2018. Version: 1.0.0).
